# Increased mitochondrial activity in a novel IDH1-R132H mutant human oligodendroglioma xenograft model: *in situ* detection of 2-HG and α-KG

**DOI:** 10.1186/2051-5960-1-18

**Published:** 2013-05-29

**Authors:** Anna C Navis, Simone P Niclou, Fred Fack, Daniel Stieber, Sanne van Lith, Kiek Verrijp, Alan Wright, Jonathan Stauber, Bastiaan Tops, Irene Otte-Holler, Ron A Wevers, Arno van Rooij, Stefan Pusch, Andreas von Deimling, Wikky Tigchelaar, Cornelis JF van Noorden, Pieter Wesseling, William PJ Leenders

**Affiliations:** 1 Department of Pathology, Radboud University Nijmegen Medical Centre, PO Box 9101, Nijmegen, 6500 HB, The Netherlands; 2 Centre de Recherche Public de la Santé (CRP-Santé), Department of Oncology, NorLux Neuro-Oncology Laboratory, Luxembourg, Luxembourg; 3 Department of Radiology, Radboud University Nijmegen Medical Centre, Nijmegen, The Netherlands; 4 IMABIOTECH, Loos, France; 5 Department of Laboratory Medicine, Radboud University Nijmegen Medical Centre, Nijmegen, The Netherlands; 6 Department of Neuropathology, Institute of Pathology, Ruprecht-Karls-University Heidelberg, Im Neuenheimer Feld 224, Heidelberg, 69120, Germany; 7 Clinical Cooperation Unit Neuropathology, German Cancer Institute (DKFZ), Im Neuenheimer Feld 224, Heidelberg, 69120, Germany; 8 Academic Medical Centre, Department of Cell Biology and Histology, University of Amsterdam, Amsterdam, The Netherlands; 9 Department of Pathology, VU University Medical Center, Amsterdam, The Netherlands

**Keywords:** Glioma, IDH mutations, Xenograft, D-2-hydroxyglutarate, α-ketoglutarate, Mitochondria: LESA-nano ESI-FTICR

## Abstract

**Background:**

Point mutations in genes encoding NADP^+^-dependent isocitrate dehydrogenases (especially *IDH1*) are common in lower grade diffuse gliomas and secondary glioblastomas and occur early during tumor development. The contribution of these mutations to gliomagenesis is not completely understood and research is hampered by the lack of relevant tumor models. We previously described the development of the patient-derived high-grade oligodendroglioma xenograft model E478 that carries the commonly occurring *IDH1-R132H* mutation. We here report on the analyses of E478 xenografts at the genetic, histologic and metabolic level.

**Results:**

LC-MS and *in situ* mass spectrometric imaging by LESA-nano ESI-FTICR revealed high levels of the proposed oncometabolite D-2-hydroxyglutarate (D-2HG), the product of enzymatic conversion of α-ketoglutarate (α-KG) by IDH1-R132H, in the tumor but not in surrounding brain parenchyma. α-KG levels and total NADP^+^-dependent IDH activity were similar in *IDH1*-mutant and -wildtype xenografts, demonstrating that *IDH1*-mutated cancer cells maintain α-KG levels. Interestingly, *IDH1*-mutant tumor cells *in vivo* present with high densities of mitochondria and increased levels of mitochondrial activity as compared to *IDH1*-wildtype xenografts. It is not yet clear whether this altered mitochondrial activity is a driver or a consequence of tumorigenesis.

**Conclusions:**

The oligodendroglioma model presented here is a valuable model for further functional elucidation of the effects of *IDH1* mutations on tumor metabolism and may aid in the rational development of novel therapeutic strategies for the large subgroup of gliomas carrying *IDH1* mutations.

## Background

Diffuse gliomas are notoriously difficult to treat, and remain incurable to this date. Based on WHO guidelines, these tumors are categorized in grade II-IV, grade IV (glioblastoma) being the most aggressive subtype [1]. Grade IV gliomas may develop *de novo* (primary glioblastoma) or evolve from lower grade tumors (secondary glioblastoma) [2-5]. The role of specific tumor suppressor genes and oncogenes in gliomagenesis has rapidly been elucidated in the last decades [6-12]. A recent breakthrough was the discovery of the involvement of mutations in the genes for isocitrate dehydrogenase 1 (*IDH1)* and, less frequently, *IDH2*[13-15] in grade II/III gliomas and secondary glioblastomas. *IDH* mutations are uncommon in other tumor types, with the exception of acute myeloid leukemia (AML), angioimmunoblastic T-cell lymphomas, intrahepatic cholangiocarcinomas and chondrosarcomas [13,16-19]. Mutations in glioma almost always involve an arginine-to-histidine conversion at position 132 in the catalytic site of IDH1 [14,20,21].

The high frequency of mutations in *IDH1* suggests an important role for the mutant protein in early glioma development, but the exact underlying oncogenic mechanism is not completely understood. IDH1 is a cytoplasmic enzyme that converts isocitrate to α-ketoglutarate (α-KG), with simultaneous reduction of NADP^+^ to NADPH [22]. The other family members IDH2 and −3 reside in mitochondria and are either NADP^+^- (IDH2) or NAD^+^-dependent (IDH3). For as yet unknown reasons, the occurrence of *IDH* mutations in glioma is restricted to the NADP^+^ dependent variants [23].

The *R132H* mutation equips the enzyme with a neomorphic activity resulting in a reduction of α-KG to D-2-hydroxyglutarate (D-2HG) [24], an NADPH consuming process. D-2HG may be an ‘oncometabolite’, but whether and how it contributes to gliomagenesis is a matter of debate. Patients with D-2-hydroxyglutaric aciduria, a rare metabolic condition in which high levels of D-2HG occur due to mutations in the D-2HG dehydrogenase gene *D2HGDH* or in *IDH2*, do not show increased levels of tumor development [25]. Paradoxically, the metabolic condition L-2-hydroxyglutaric aciduria has been suggested to predispose for brain tumorigenesis [26,27]. Depletion of α-KG, an essential citric acid cycle intermediate, in IDH1 mutant tumor cells may play a role during tumor development [28] but competitive inhibition by D-2HG of the 60 known α-KG-dependent enzymes appears to be a more important factor [29]. For example, inhibition of α-KG-dependent TET2 and histone demethylase KDM4C (also known as JMJD2C) results in DNA hypermethylation and histone demethylation respectively and, as a result, blocks cell differentiation [30-32]. Furthermore, increased degradation of hypoxia-inducible factor 1α (HIF1α) via inhibition of α-KG dependent EGLN prolyl 4-hydroxylases may play a role [33]. Recently, it was described that this mechanism contributes to leukemogenesis in a reversible manner [34].

*IDH1* mutations nearly always occur in a heterozygous fashion and the presence of a wild type (wt) allele is required for the maximal neomorphic activity of the mutant protein [35]. The enzyme consists of two independently acting subunits. Wild type enzyme subunits, either as homodimer or in mutant/wt heterodimers, convert isocitrate to α-KG which is subsequently processed by the mutant enzyme (subunits) to D-2HG [36]. Whereas wtIDH1 can convert α-KG back to isocitrate in a CO_2_- and NADPH-dependent manner, this activity is lost by the R132 mutation [22]. The stoichiometry of IDH1 wt and mutant proteins is likely an important parameter which determines α-KG and D-2HG levels and may therefore influence cell metabolism [35]. Therefore, it is not clear to what extent exogenous introduction of recombinant mutant IDH1 in cell lines provides relevant information as overexpression may result in a non-physiological amount of IDH1-mutant homodimers which may differ in activity from heterodimers [37]. Moreover, metabolism of cells under standard culture conditions may significantly differ from *in vivo* conditions where local areas of hypoxia and hypoglycemia routinely occur. Therefore, it is important to study relevant orthotopic glioma xenograft models that recapitulate the biology of tumors carrying the endogenous mutation. It has been shown by several groups that gliomas with IDH1 mutations are difficult, if not impossible, to culture and propagate *in vitro* under standard serum-free or serum-containing culture conditions [38-40]. Similarly, *in vivo* models are difficult to propagate and as a result, preclinical glioma models carrying the IDH1 or IDH2 mutation are scarce.

Here, we report on the genetic, histologic and metabolic characterization of the E478 human oligodendroglioma xenograft line which carries the endogenous heterozygous IDH1-R132H mutation and provide novel insight into the metabolism of these tumors.

## Results

### Development of IDH1-R132H xenografts

In our institute, we have a long history of developing patient-derived orthotopic glioma xenograft models by direct intracerebral implantation of cancer cell suspensions from surgically-obtained glioma specimens [41]. As long-term *in vitro* cell cultures are known to be genetically unstable [42], the development of such direct orthotopic xenografts is important in the context of clinical relevance and reproducibility. Remarkably, from 5 biopsies derived from IDH1-R132H-mutated high-grade oligodendroglioma specimens only one so far gave rise to the stable xenograft line described here (E478). This is in line with a previous report which demonstrated that xenografting of cultured IDH1 mutant glioma cells hardly results in *in vivo* tumor growth [38] and is in sharp contrast with our experience with orthotopic xenografting of IDH1wt gliomas in which success rates approach 100%. Attempts to culture E478 cells *in vitro* using both neurosphere and standard culture conditions were so far unsuccessful (data not shown). Occasionally, we managed to maintain short-term organotypic spheroid cultures [43] and these were used for genetic analyses (Additional file 1 and Additional file 2: Figure S1).

To increase the versatility of the E478 model, we generated cell suspensions directly from xenografts and cryopreserved these before re-injecting them intracerebrally in mice. This procedure resulted in successful orthotopic engraftment in 100% of the animals, also after re-transplantation.

**Figure 1:**
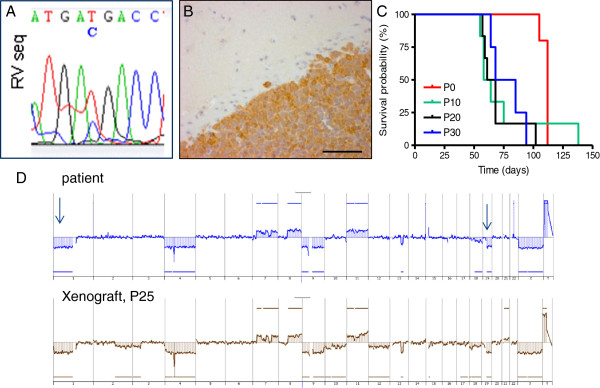
**E478 xenografts contain the IDH1-R132H mutation. A**) E478 xenografts contain the G=> A mutation as shown by direct sequencing. **B**) Micrograph of a E478 xenograft in mouse brain, stained with the IDH1-R132H-specific antibody, bar =100 μm. **C**) Curves of time to sacrifice of mice in various passages of the E478 line, P0 being the first transplantation of surgically-obtained tumor material, directly from the patient in nude mice. **D**) Comparison of array CGH profiles of the tumor of the patient (blue) and a derived xenograft (P25, brown). The losses of chromosome arms 1p and 19q, typical for oligodendroglioma, are indicated by arrows in the profile of the patient tumor. All genetic aberrations that were present in the original tumor, are present in the profile of the xenografts as well, with a few additional aberrations in the xenograft. Further details are presented in Additional file 2: Figure S1.

The E478 xenograft model has now been maintained in the brains of Balb/c *nu/nu* mice by serial transplantation for over 32 passages (P) in a period of over 8 years. PCR sequencing of *IDH1* confirmed the maintenance of the heterozygous *c.395G > A* mutation [NM_005896.2] resulting in the R132H conversion in IDH1 (Figure 1A), similarly to the parental tumor (data not shown). Cytoplasmic expression of the mutant IDH1 protein was readily detected in E478 xenografts using IHC and a monoclonal antibody that specifically recognizes the R132H mutated IDH1 protein [44] (Figure 1B). Tumor take of E478 xenografts after intracerebral passaging is over 95% and the median time until mice are sacrificed because of tumor-related symptoms is 78 days (n = 200, P 0, 10 and 20 shown in Figure 1C).

### Genetic analysis of E478 xenografts

To compare the chromosomal aberrations in the E478 xenografts and the parental tumor, we performed array comparative genomic hybridization (aCGH) analyses (Figure 1D). The original tumor showed complete hemizygous loss of chromosomal arms 1p and 19q, a characteristic feature of oligodendroglial tumors. Additionally, hemizygous losses of chromosomes 4, 9 as well as 13q21.33-31.2 were detected. A region in chromosome 4 (59.5-62.2 Mb), which is devoid of any known genes or miRNAs, was homozygously deleted. Furthermore, the tumor was triploid for 7p15.2-qter, 8q12.3-qter and 11 (Figure 1D, upper panel). All aberrations were maintained in late passages (Figure 1D, lower panel). Some additional aberrations were also detected in the xenograft, including loss of 2q22-qter, 3pter-p21.2, chromosomes 10, 12 and 18 as well as gains of chromosome 21 (Additional file 2: Figure S1). Glial tumors show a high level of intratumoral genomic heterogeneity [45] which might explain the differences observed between the original tumor biopsy and its derived xenograft.

We determined the DNA quantity per cell of the xenograft tumor by flow cytometry of DAPI-labeled nuclei (Additional file 3: Figure S2A). A minor fraction of the cells appeared to be diploid and was considered to consist of stromal or host-derived cells. The majority of cells were aneuploïd with a DNA index of 1.925, which corresponds to 3.85 N. The xenograft tumor thus has a near tetraploïd genome with some xenograft-specific losses. The inferred copy number at the IDH1 locus on 2q34 is 3.

In order to determine the genotype at the IDH1 locus, we set up an allele-specific TaqMan SNP genotyping assay that can discriminate between the wild type and R132H alleles of *IDH1*. This assay revealed that the *R132H* allele was twice more abundant than the wt allele in the xenograft (Additional file 1 and Additional file 3: Figure S2B), resulting in an IDH1^R132H/R132H/WT^ genotype in the xenograft.

### Phenotype of the E478 xenografts

**Figure 2:**
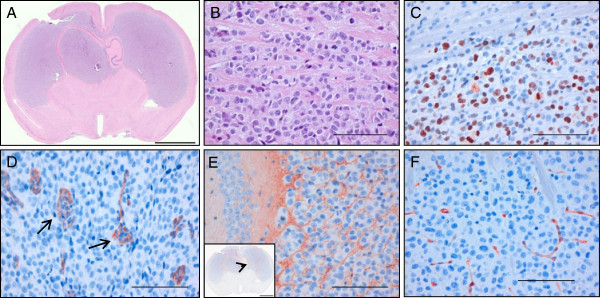
**Phenotypic characteristics of the IDH1 mutant xenograft model.** Low (**A**) and high (**B**) magnifications of H&E-stained sections of E478 xenografts in mouse brain, showing diffuse infiltration throughout both hemispheres (note that infiltrative strands of cancer cells are interspersed in white matter [B]). **C**) Ki-67 staining resulted in a proliferation index of approximately 34%. **D**) Immunohistochemical anti-CD34 staining shows abundant presence of florid microvascular proliferations. **E**) Immunostaining of mouse IgG shows limited and focal leakage of IgG from the tumor vasculature. The arrowhead in the low-magnification inset indicates the area depicted. **F**) Blood vessels in the tumor express GLUT-1 that is characteristic for endothelial cells forming the blood–brain barrier. Cancer cells do not express GLUT-1, indicating that the tumor is not hypoxic. Size bars in A: 1 mm, B-F 100 μm.

E478 xenografts characteristically grow to diffuse infiltrative tumors in the mouse brain (Figure 2A,B) with proliferation indices of 34 +/− 2% as determined by Ki67 IHC staining (Figure 2C and not shown). Apoptotic cells, as determined by IHC for activated caspase 3A, were hardly detected (<0.1%; data not shown). Blood vessels were often abnormal with signs of endothelial hyperplasia and microvascular proliferation with prominent CD34 staining (Figure 2D), reminiscent of typical high grade glioma pathology. The presence of extravascularly deposited mouse IgG indicated focal disruption of the blood brain barrier (BBB; Figure 2E). The tumor vasculature was positive for the BBB-marker GLUT-1 throughout the tumor (Figure 2F). However, tumor cell-associated GLUT-1 or monocarboxylate transporters MCT1 and MCT4 were not present (Figure 2F and data not shown). The absence of these HIF1α-regulated hypoxia markers is in accordance with recent studies that show an inhibition of HIF1α expression induced by D-2HG and EGLN in *IDH1*-mutant tumor cells [33,34].

### D-2HG production in E478 xenografts

**Figure 3:**
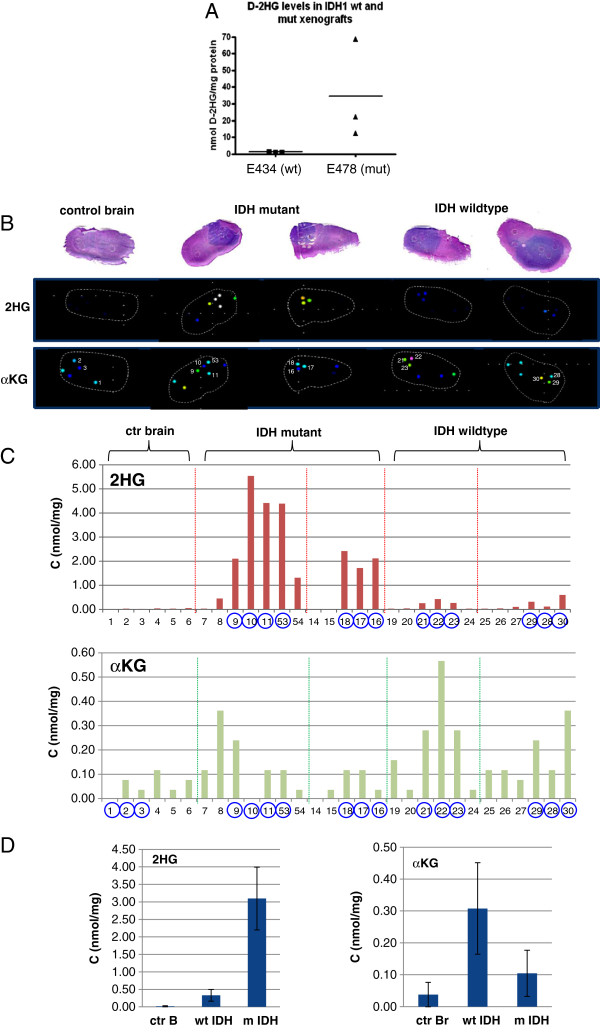
**D-2HG and α-KG levels in xenografts. A**) D-2HG levels in extracts of xenograft-containing mouse brains from E478 *IDH1*-mutant and E434 *IDH*-wild type (wt) tumors as detected by LC-MS. **B**) In situ detection of D-2HG and α-KG levels in tumor sections using LESA-nano ESI-FTICR. Upper panel shows H&E stained sections of mouse brain (pink) with tumors (blue) from which tissue plugs were analysed. Lower panels show Quantinetix views of tissue plugs for 2-HG (m/z 147.032) and α-KG (m/z 145.017) respectively. **C**) Quantification of D-2HG and α-KG levels in individual tissue plugs taken from control brain, *IDH1*-mutant xenografts and *IDH*-wt xenografts. Sample numbers encircled in blue represent tissue plugs as shown in (**B**). **D**) Mean D-2HG and α-KG levels in control brain (ctr B), *IDH1*-mutant xenografts (m IDH) and *IDH*-wildtype xenografts (wt*IDH*). Note the difference in scale of the Y-axes in these graphs. Levels of α-KG were very low as compared to D-2HG levels, but clearly detectable in all plugs.

D-2HG levels were determined in extracts of E478 xenografts using liquid chromatography coupled to mass spectrometry (LC-MS) [24]. E478 tumor extracts contained highly elevated levels of D-2HG (34.5 nmol/mg protein vs < 0.2 nmol/mg protein in *IDH1*-wt E434 xenografts; n = 3, p < 0.0001; Figure 3A). D-2HG levels in plasma of tumor-bearing mice were not altered (data not shown) which is in agreement with the recent finding that plasma levels of D-2HG do not correlate with *IDH1*-mutation status in glioma patients [46].

D-2HG levels in the brain extracts used in our LC-MS measurements varied highly (Figure 3A), a likely consequence of variations in tumor/stroma ratios in the extracts. To more reliably determine spatial D-2HG production in E478 xenografts, we used Quantinetix technology which allows sensitive and quantitative measurements of metabolites *in situ* in tissue sections using Liquid Extraction Surface Analysis (LESA) coupled to High Resolution Electrospray Mass Spectrometry (HR-ESI-MS) (Figure 3B). Our measurements were performed in spots with a size of 0.12 mm^2^. Concentrations were expressed as heat maps, superimposed on histologically stained sections (Figure 3B). D-2HG levels were increased in tumor tissue only (60–820 μg D-2HG/g tissue; Table 1), while much lower concentrations of the metabolite were detected in non-tumor brain areas and in healthy mouse brain. In line with the LC-MS measurements, the difference in D-2HG levels between *IDH1*-mutant tumors and normal brain was over 100-fold (3.1 vs 0.02 nmol/mg; Table 1). We also found significantly elevated levels of D-2HG in *IDH1-*wt glioblastoma xenografts as compared to normal brain, although the levels were lower than in *IDH1*-mutant tumors (approximately 10-fold difference: 3.1 vs 0.33 nmol/mg). Indeed, it has been shown that wtIDH1 can convert α-KG to D-2HG. However, this reaction is hampered by competitive displacement of α-KG by isocitrate in the catalytic site under normal conditions [36]. A low isocitrate/α-KG ratio may thus result in D-2HG production as well.

**Table 1:** D-2HG and αKG levels in individual spots in sections of othotopic xenografts carrying the IDH1-R132H mutation (italic) or wild-type IDH

***Spot number***	***D-2-HG (μg/g)***	***α-KG (μg/g)***
**1**	0.0	0
**2**	4.7	11.2
**3**	2.9	5.2
**4**	6.5	17.2
**5**	4.7	5.2
**6**	8.3	11.2
**7**	4.7	17.2
***8***	*66.7*	*52.9*
***9***	*311*	*35*
***10***	*819.8*	*0*
***11***	*653.9*	*17.2*
***53***	*650.2*	*17.2*
***54***	*194.3*	*5.2*
***14***	*0*	*0*
***15***	*0*	*5.2*
***18***	*358.4*	*17.2*
***17***	*254.5*	*17.2*
***16***	*312.9*	*5.2*
**19**	4.7	23.1
**20**	6.5	5.2
**21**	37.5	41
**22**	63	82.7
**23**	39.3	41
**24**	4.7	5.2
**25**	4.7	17.2
**26**	6.5	17.2
**27**	15.6	11.2
**28**	46.6	35
**29**	17.4	17.2
**30**	88.6	52.9

Spots 1-6 represent normal brain.

In line with previous observations [24] we detected similar levels of α-KG in all tumor samples, both IDH1-mutant and -wt. There was a tendency towards higher levels in tumors compared to normal brain, although the levels were not significantly different. These findings suggest that the α-KG pool is maintained at physiological levels despite its depletion by conversion into D-2HG in the *IDH1*-mutant tumor.

In summary we show that in *IDH1*-mutant xenografts D-2HG levels are approximately 100-fold higher as compared to normal brain tissue and that these high levels are restricted to the tumor area. Moreover, α-KG levels are maintained at physiological levels in these tumors.

### NAD^+^- and NADP^+^-dependent dehydrogenase activity in E478 tumors

**Figure 4:**
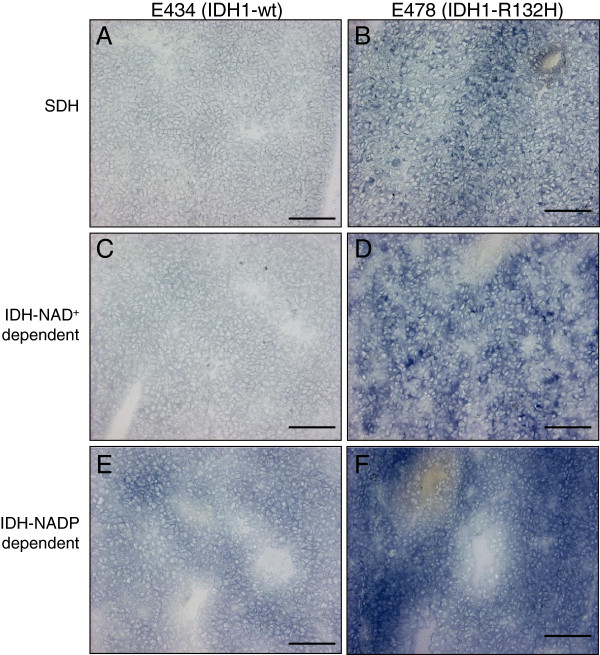
**Metabolic mapping.** Localization of activity of SDH (**A,B**), NAD^+^-dependent IDH (**C,D**) and NADP^+^-dependent IDH (**E,F**). The blue color represents the activity of the respective dehydrogenases after 15 minutes of incubation at 37°C. Note the high activities of mitochondrial dehydrogenases in E478 xenograft as compared with the E434 xenograft. Bar = 100 μm.

IDH1-R132H expression is expected to result in diminished levels of α-KG in the cytosol. Since we did not observe detectable decreases in total cellular a-KG levels, we argued that E478 cells may depend on mitochondrial IDHs to generate α-KG for cytosolic use. To test this, 10 μm-thick cryostat sections of E478 and E434 oligodendroglioma xenografts were subjected to metabolic mapping to stain activity of NAD^+^- and NADP^+^-dependent IDHs and succinate dehydrogenase (SDH), as a read-out for mitochondrial activity. While E434 xenografts showed low activity of SDH and NAD^+^-dependent IDH (Figure 4A,C), the activities of these enzymes were much more prominent in E478 xenografts (Figure 4B,D). Although NADP^+^-dependent IDH activity was higher than NAD^+^-mediated IDH activity in both xenografts (compare E,F to C,D), this increase was far more distinct in E478 xenografts (Figure 4F). The high NADP^+^-dependent IDH activity in E478 suggests that the cancer cells compensate the IDH1 defect by upregulating the activity of the mitochondrial IDH2.

**Figure 5:**
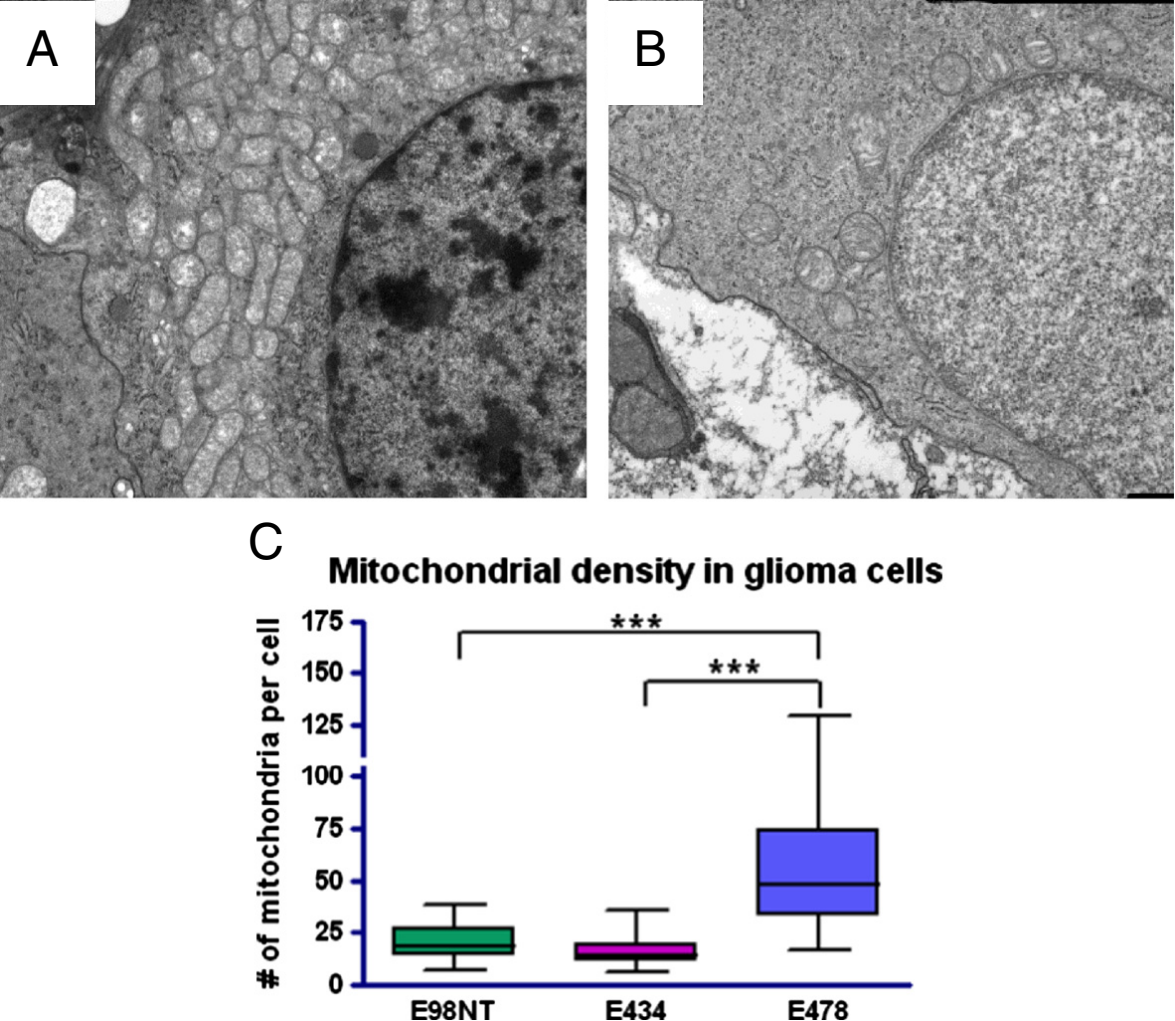
**Transmission electron microscopy.** Transmission electron microscopical micrographs of the increased mitochondrial density in E478 (**A**) as compared to E434 xenografts (**B**). **C**) Quantification of mitochondrial densities in IDHwt E98 and E434 xenografts and IDH1-R132H E478 xenografts).

To further investigate the high mitochondrial activity in E478, we performed transmission electron microscopy on a panel of glioma xenografts. Measurement of mitochondrial densities revealed a 2-fold increase in the number of mitochondria in the IDH1-mutant E478 tumor cells as compared to those in E434 anaplastic oligodendroglioma and the E98 glioblastoma xenograft lines (Figure 5A-C).

## Discussion

We here present a detailed histologic and metabolic characterization of the E478 *IDH1-R132H* mutant oligodendroglioma xenograft line, the development of which has been described before [41]. This line was established already in 2005, before the recognition of the involvement of *IDH1/2*-mutations in gliomagenesis. The E478 xenograft line has been grown successfully for over 35 passages now, is genetically stable and produces elevated levels of the oncometabolite D-2HG.

It is still not completely understood how IDH1 mutations in gliomas contribute to tumorigenesis and at the same time are correlated to good prognosis as compared to gliomas with wt*IDH*[21,47]. Likely, the effects are multifactorial. Elevated D-2HG levels have been proposed to result in a block of differentiation via epigenetic alterations [30], including the induction of a hypermethylated DNA phenotype [35]. Hypermethylation of genes encoding DNA repair enzymes such as *MGMT* is a predictor for the response to alkylating chemotherapy [48]. However prognosis of patients with *IDH1* mutated glioma is not confined to those receiving chemotherapy, suggesting that other factors may also be important. Models such as the E478 xenograft may contribute to elucidate the underlying mechanisms of IDH mutations with respect to gliomagenesis and prognostic relevance. Our data indicate that some answers may be found in the field of tumor metabolism as we observed mitochondrial hyperactivity in E478 xenografts. It remains unfortunate that only a limited number of endogenous *IDH1-R132H* mutant glioma models is currently available [38,40] making it difficult to confirm our findings in other models.

We found that NADP^+^-dependent isocitrate conversion was not decreased in E478 xenografts as compared to *IDH*wt glioma, indicating that cells compensate for loss of IDH1 activity by increasing mitochondrial IDH2 activity by inducing mitochondrial biosynthesis. This is in agreement with our finding that α-KG levels are not diminished in E478 xenografts as compared to *IDH*wt tumors. These data suggest that D-2HG, rather than a shortage of α-KG, is involved in the specific features of *IDH1*-mutated glioma and is in line with D-2HG-mediated competition of α-KG dependent enzymes. Yet, our measurements do not allow discrimination between mitochondrial and cytosolic α-KG levels, and a role for a specific depletion of the cytosolic pool of α-KG in tumor biology cannot be excluded. For instance, fatty acid synthesis is an essential condition for tumor growth, and requires cytosolic α-KG and acetyl-CoA as precursors [49]. Depletion of cytosolic α-KG may compromise lipid biosynthesis and might require import of mitochondrial α-KG into the cytosol. An attractive hypothesis is that such processes require a lot of energy for cell survival at the expense of extensive tumor cell proliferation. This may be a possible explanation for the better prognosis of patients with *IDH*-mutated gliomas.

An alternative manner to deal with decreased cytosolic α-KG, apart from increasing IDH2 activity, may be increased import of glutamine via specific importers in glioma cells. Glutaminase converts glutamine to glutamate which is subsequently converted to α-KG by glutamate dehydrogenase [50]. This metabolic adaptation may be in line with the increased dependency of *IDH1*-mutant gliomas on exogenous glutamine, the low levels of glutamate in *IDH1*-mutant gliomas [51] and the sensitivity of these tumors to glutaminase inhibitors [52]. The relative contribution of these systems in α-KG homeostasis remains to be determined.

It is still enigmatic why mutations in *IDH* genes are only found in the NADP^+^-dependent enzymes. It is tempting to speculate that this is related to a specific aspect of NADP^+^ metabolism. During conversion of α-KG to D-2HG by IDH1-R132H, NADPH is oxidized to NADP^+^ and is not available for generation of reduced glutathione and other detoxifying systems [53]. As reduced glutathione is essential for scavenging reactive oxygen species (ROS), this may result in increased oxidative stress in *IDH1*-mutant glioma cells, a phenomenon that will be further augmented by increased mitochondrial density. Indeed, glutathione levels appear to be lower in *IDH1*-mutated tumors [51]. How *IDH1*-mutant tumors cope with this stress is currently under investigation in our laboratory.

Based on our findings we propose that tumor cells that carry the *IDH1* mutation undergo a metabolic switch involving increased mitochondrial activity leading to impaired proliferation and a relatively good prognosis. Cells acquiring *IDH1* mutations will become tumorigenic based on the oncogenic activity of D-2HG, possibly in combination with acquired mutations in *TP53*[34,54]. Acquiring *IDH1* mutations comes at a price however, as it will pose the cell with an excessive need for exogenous sources of α-KG for sustained membrane synthesis. The increased requirement for α-KG results in an increased dependency on exogenous glutamine. Interestingly, glutamine is produced at high levels by glial cells and is under normal conditions used by neurons as a precursor for the neurotransmitter glutamate. It would be interesting to investigate whether the dependency on glutamine or glutamate as external carbon source has a causal relation to the diffuse infiltrative growth in neuroglial tissue that is characteristic of diffuse gliomas. Indeed, such a dependency suggests that tumor cells would benefit from the nearby presence of non-neoplastic glial cells or neurons.

This model thus provides novel handles for metabolic targeting of low grade gliomas. Especially inhibition of glutaminolysis may be an effective way to interfere with glioma metabolism, although the effects of such an intervention on neuronal glutamate function will require careful evaluation. Combination approaches to further increase redox stress and/or glycolysis may further arrest tumor cells.

## Conclusions

The E478 xenograft line represents a stable tumor model with the endogenous IDH1-R132H mutation. We show that this model has high mitochondrial activity, produces high levels of D-2HG and maintains near-physiological levels of α-KG. We propose that this model will be of high value for investigating novel therapies for the large group of gliomas that carry *IDH1-R132H* mutations.

## Methods

### Intracranial xenografting

Athymic female BALB/c nu/nu mice (18–25 gram, age 6–8 weeks) were kept under specified pathogen-free conditions and received food and water *ad libitum*. The local Animal Experimental Committee of the Radboud University Nijmegen Medical Centre approved all experiments. Glioma cell suspensions, directly obtained from surgically resected tumor tissue of a patient with an anaplastic oligodendroglioma, which was later shown to carry the IDH1-R132H mutation, were injected intracranially as described previously [41]. Animals were closely monitored and sacrificed when evident signs of tumor burden (especially weight loss >15% in two days, severe neurological abnormalities) were observed. The xenograft line has been maintained by direct intracerebral passaging of tumor cell suspensions, generated from E478-bearing mouse brains as described [41].

### Cryopreservation of xenograft brains

To test whether E478 cancer cells retain their tumorigenic potential upon cryopreservation, cell suspensions of tumor-bearing brains were generated in PBS and washed twice followed by suspension in DMEM (containing 0.45% w/v glucose; PAA Laboratories, Pasching, Austria) with 5% DMSO (Merck, Nottingham, UK). Cell suspensions were frozen at −1°C/min in Nalgene Mr. Frosty containers (ThermoScientific, Landsmeer, The Netherlands) at −80°C and subsequently stored in liquid nitrogen for at least a month. For re-injection, cells were rapidly thawed at 37°C, washed twice and suspended in PBS, followed by intracranial injection in mice (n = 5) as described above. When animals developed tumor-related symptoms, a tumor of one of the mice was transplanted into new animals (n = 5).

### Array comparative genomic hybridization (aCGH)

aCGH was carried out with DNA of the originating patient tumor and several passages of the derived xenografts. DNA was digested using the restriction enzymes RsaI and AluI, followed by labeling using the Bio Prime CGH Genomic Labeling Kit (Invitrogen, Carlsbad, CA) and Cy3 and Cy5 dyes (GE Healthcare, Buckinghamshire, UK), according to standard protocols for Agilent CGH. Commercially available female DNA pooled from multiple donors (Promega Cat:G1521) was used as reference. Labeled DNA was competitively hybridized to SurePrint G3 Human 2×400k CGH microarrays (G4448A, Agilent Technologies, Amstelveen, The Netherlands) following standard protocols. The slides were scanned at 3 μm resolution using the Agilent High-Resolution Microarray scanner and the image data were extracted with Feature Extraction software (Agilent Technologies). Feature extraction files were imported into Genomic Workbench 7.0 for visualization and analysis. Briefly, after diploid centralization and GC correction, aberrations were called using the ADM2 algorithm with a threshold setting of 20, centralization on with a threshold of 25 and an aberration filter min Probes = 5 and minAvgAbsLogRatio = 0.25 for amplifications and deletions.

### Immunohistochemistry (IHC)

Animals with tumor-related symptoms were sacrificed by cervical dislocation and brains were removed for fixation in buffered formalin and subsequent paraffin embedding. Parts were also snap frozen in liquid nitrogen and stored for protein analysis and DNA/RNA isolation, D-2HG measurements and metabolic mapping.

H&E and immunohistochemical stainings were performed as described previously [55] including the use of antibodies directed against IDH1-R132H (clone H09, Dianova, Hamburg, Germany), Ki67 (for proliferation index assessment, clone Sp6, Neomarkers, Fremont, CA), cleaved caspase 3A (for detection of apoptotic cells, clone C92-605, BD Pharmingen, Franklin Lakes, NJ), CD34 (for endothelial cell staining, clone MEC14.7, Hycult Biotech, Uden, The Netherlands), GLUT-1 (Neomarkers), mouse IgG (Vector, Burlingame, CA), MCT-1 and MCT-4 (clones C-20 and H-90 respectively, Santa Cruz, CA). Primary antibody incubations were performed using 4 μm-thick sections of formalin-fixed paraffin-embedded tumor samples. Appropriate biotinylated secondary antibodies were used for detection using the ABC-method (Vector Laboratories). Specific signals were visualized by staining with 3-amino-9-ethyl-carbazole (Scytek Laboratories, West-Logan, Ut). All sections were counterstained with haematoxylin and mounted in Imsol Mount medium (Klinipath B.V., Duiven, The Netherlands). For all stainings, control incubations were carried out by omitting the primary antibody.

### Metabolic mapping

Activities of NADPH- and NADH-producing dehydrogenases were visualized using metabolic mapping [53,56] using 10 μm thick unfixed cryostat sections of mouse brains infiltrated with E478, E434 or E98 glioblastoma xenografts [41]. The wt IDH1 status of E434 and E98 xenograft lines (oligodendroglioma and glioblastoma, respectively) was previously confirmed. Control incubations to establish the specificity of the enzyme reactions, were performed in the absence of relevant substrates [53,56].

### Electron microscopy

Tissue samples of approximately 1–2 mm^3^ were fixed in 2% glutaraldehyde in cacodylate buffer (100 mM) for 4 hours, rinsed in cacodylate buffer and post-fixed for 1 hour in a solution of 1% osmium tetroxide containing 1% potassium hexacyanoferrat. Semithin (1 μm) sections and ultrathin (70 nm) sections were cut on an ultramicrotome (Leica EM UC6). Semithin sections were stained with toluidin blue for light microscopical previewing and ultrathin sections were collected on 200 mesh copper grids and contrasted with uranyl acetate and lead citrate. All sections were examined and images generated on a JEM1200 transmission electron microscope (Jeol, The Netherlands).

### D-2HG measurements by isotope dilution LC-MS

D-2HG levels in serum and tissue extracts were measured using stable isotope dilution liquid chromatography tandem mass spectrometry (LC-MS). D-2HG for the preparation of calibration standards was purchased from Sigma Aldrich. Samples of 100 μl were mixed with 50 μl of ^13^C_5_-2-HG stable isotope solution (Chiralex, Nijmegen, The Netherlands; 10 μM in deionized water) before passing it through a Microcon YM-30 filter (Millipore) by centrifugation (14,000xg; 30 min). After acidification of the filtrate with 10 μl 4% formic acid in deionized water, 5 μl was injected into a Luna PFP column (2.1 mm*100 mm*3 μm, Phenomenex). The mobile phase consisted of methanol and water containing 0.3% formic acid. 2-HG was separated from its isomers 3-hydroxyglutarate and 2-hydroxy-2-methylsuccinate using a water-to-methanol gradient at 250 μl/min. The column was connected to an electrospray tandem mass spectrometer (Quattro LC, Micromass) operated in negative mode (capillary voltage 3 kV, cone voltage 20V) with an argon filled collision cell (0.18 Pa, 9eV). The tandem mass spectrometer was set to monitor the water loss of both D-2HG and ^13^C_5_-2-HG recording the mass transitions of m/z 147 to 129 and m/z 152 to 134, respectively. The temperature settings for the source and ion block were 400°C and 100°C respectively. Nitrogen was used as drying and nebulizer gas set at flow rates of respectively 650 L/h and 100 L/h.

### *In situ* metabolite quantification by LESA-nano ESI-FTICR

In a separate set of experiments, we quantified D-2HG and α-KG levels via LESA (Liquid Extraction Surface Analysis, Nanomate, Advion) coupled to ESI-FTICR (Fourier transform ion cyclotron resonance, Solarix 7T, Bruker Daltonics, Bremen) and Quantinetix Software (ImaBiotech, France) allowing very sensitive detection and quantification of metabolites in small tissue plugs taken from 10 μm thick cryostat brain sections (Cryostat HM560, Microme). The Nanomate system was used in LESA mode with a 400 μm diameter surface extraction. Sample plates were cooled down to 12°C during analyses. Spray parameters were set as follows: Voltage to apply 1.30 kV and gas pressure 0.40 psi. Extraction solvent consisted of 65:15:20 MeOH:IPA:Water + 5 mM ammonium acetate using LCMS quality solvents. 0.6 μL extraction solvent was used to extract analytes from the surface and was injected then into the nano-electrospray source. Tissue suppression was calculated using Quantinetix to address matrix effect and to normalize signals of 2-HG and α-KG. A dilution range of pure metabolites mixed with brain tissue was applied next to the samples for absolute quantification and data for each LESA spot were analyzed using Quantinetix™ (ImaBiotech, France) taking 10 mDa mass tolerance around theoretical *m/z* of 2-HG and α-KG. Data were normalized against a standard included in the extraction solvent (*m/z* 141.019). Mass spectrometry was performed using the negative mode with the nano-electrospray source and CASI mode (isolation of *m/z* 150 +/− 50 Da in the quadrupole) in the mass range 71–160 Da. Each acquisition was a result of 80 accumulated spectra.

## Competing interests

The authors declare that they have no competing interests.

## Authors’ contributions

ACN and WPJL designed the experiments and wrote the manuscript, SPN, FF, DS and JS performed array and polyploidy analyses, qPCR and LESA-nano ESI-FTICR and contributed to the writing of the manuscript. SvL, BT, AvD, PW and AW contributed significantly to the discussion, KV and SP performed immunostainings and sequence analyses, IO performed EM analysis, RAW and AR were responsible for LC-MS analysis and WT and CJFvN performed the enzymatic mapping assays. All authors read and approved the final manuscript.

## Supplementary Material

Additional file 1 Supplementary data. Click here for file

Additional file 2: Figure S1 Array CGH of E478 xenografts, short term E478 spheroid cultures and the original patient tumor. Click here for file

Additional file 3: Figure S2 Ploidy analysis of E478 xenografts (**A**) and allele-specific qPCR (**B**), showing that E478 cells contain two copies of the IDH1-R132H allele and one wild type allele. Click here for file
