# Supplementary material for: Increased mitochondrial activity in a novel IDH1-R132H mutant human oligodendroglioma xenograft model: in situ detection of 2-HG and α-KG
Source: Acta Neuropathol Commun. 2013 May 29;1:18. doi: 10.1186/2051-5960-1-18 (PMC3893588; doi:10.1186/2051-5960-1-18)
Supplement: Additional file 1 — Supplementary data. [file 2051-5960-1-18-S1.doc]

**Supplementary data**

**Materials and Methods**

***Ploidy analysis***

Xenograft tissue was minced in NST buffer [146 mM NaCl, 10 mM Tris-HCl (pH 7.5), 0.2% Nonidet P40] containing DAPI. Nuclei were disaggregated by repetitively passing the minced biopsies sequentially through a 20G and a 25G needle, followed by sequentially passing through 50-μm and 30-μm mesh filters. Flow cytometry was carried out using a BD Biosystems Aria II flow cytometer with UV excitation and DAPI emission collected at >450 nm. DNA content and cell cycle were analyzed using ModFitLtsoftware (VSH).

***Allele-specific PCR***

Allele-specific PCR was carried out using a custom TaqMan SNP Genotyping assay (Life Technologies) following the manufacturers’ instructions. Quantitative PCR reactions were carried out in triplicate in a reaction volume of 25 µl in a Viia7 Instrument (Life Technologies) with the following thermal cycler conditions: 10’ at 95°C each of 40 cycles of 15’’ at 92°C and 1’ at 60°C. DNA quantity was 20 ng per reaction. Primers used for amplification were: Forward 5’-CTTGTGAGTGGATGGGTAAAACCTA-3’, Reverse 5’-CCAACATGACTTACTTGATCCCCATA-3’. Taqman probes were: IDH1WT CATCATAGGTCGTCATGC (VIC) and IDH1R132H ATCATAGGTCATCATGC (FAM). All primers were human specific and do not amplify mouse sequences.

**Supplementary Figure S1.**

Detailed comparative array CGH analysis of the original anaplastic oligodendroglioma of the patient, E478 xenografts (P24) and a short-term spheroid culture derived from E478 (P25). Some losses were present in the xenograft which were not recognized in the original tumor of the patient (arrows). The magnitude of these losses was at a Log^2^ ratio of -0.35 (dotted line) as compared to an average Log^2^ ratio of -0.8 for the losses inherited from the originating tumor (dashed line). This corresponds to a relative copy number of 0.75 and 0.5, respectively. The 0.75 ratio originates from the tetraploid tumor background.

**Supplementary Figure S2.**

A) Ploidy analysis of E478 xenografts. Two cell cycles are detected, the diploid peak (red) corresponds to the host-derived mouse cell fraction, the aneuploid (3.86N) peak (yellow) corresponds to the human cancer cell fraction. B) Allele-specific quantitative PCR discriminating wild type and IDH1-R132H mutant alleles. The quantity of IDH1-R132H is double that of the IDH1-wild type allele.
