# Supplementary figures and images for: Increased mitochondrial activity in a novel IDH1-R132H mutant human oligodendroglioma xenograft model: in situ detection of 2-HG and α-KG
Source: Acta Neuropathol Commun. 2013 May 29;1:18. doi: 10.1186/2051-5960-1-18 (PMC3893588; doi:10.1186/2051-5960-1-18)

## Slide 1
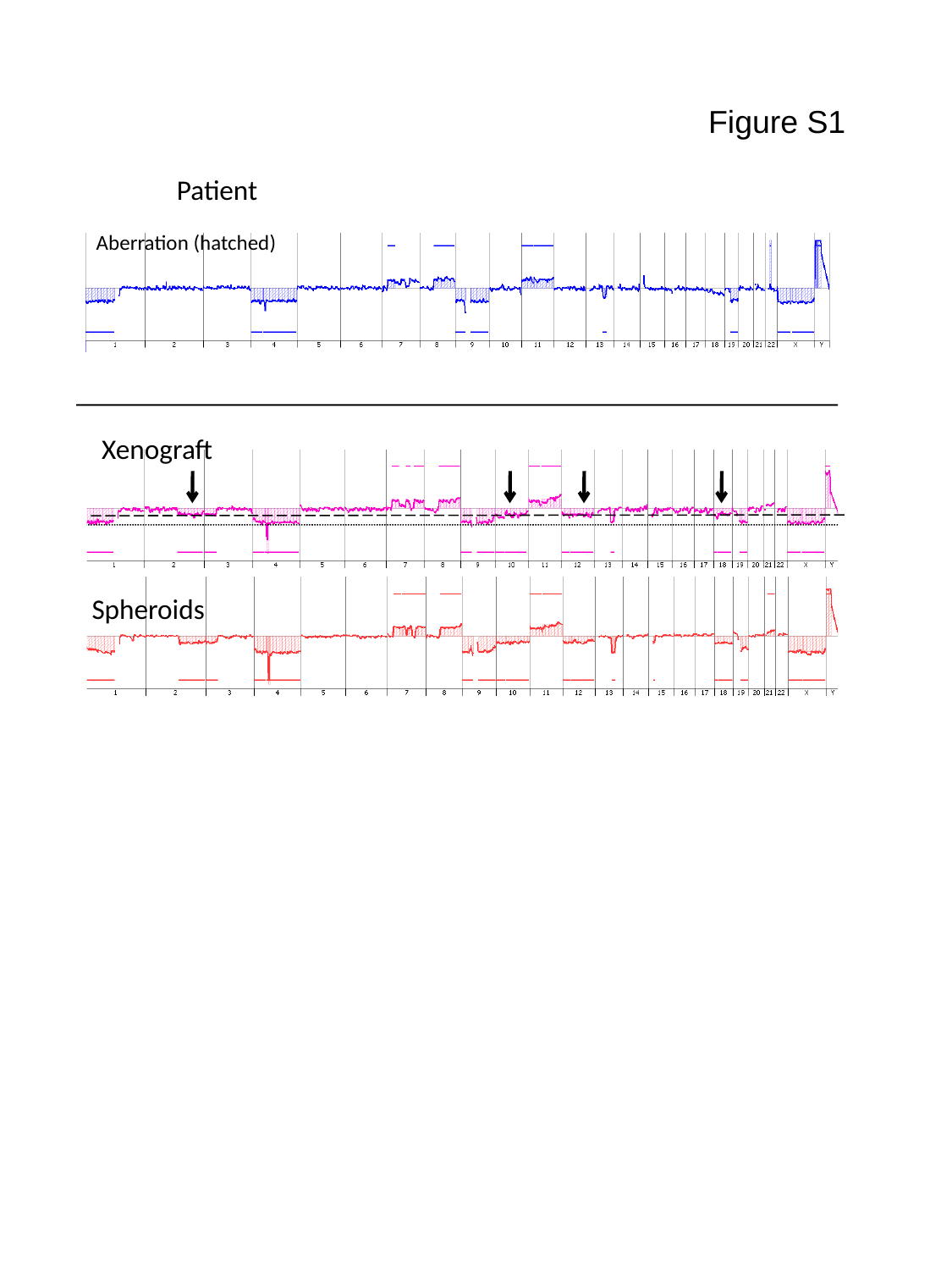

Figure S1
Patient
Aberration (hatched)
Xenograft
Spheroids

Supplement: Additional file 2: Figure S1 — Array CGH of E478 xenografts, short term E478 spheroid cultures and the original patient tumor. [file 2051-5960-1-18-S2.ppt]
