# Supplementary material for: Increased mitochondrial activity in a novel IDH1-R132H mutant human oligodendroglioma xenograft model: in situ detection of 2-HG and α-KG
Source: Acta Neuropathol Commun. 2013 May 29;1:18. doi: 10.1186/2051-5960-1-18 (PMC3893588; doi:10.1186/2051-5960-1-18)
Supplement: Additional file 3: Figure S2 — Ploidy analysis of E478 xenografts (A) and allele-specific qPCR (B), showing that E478 cells contain two copies of the IDH1-R132H allele and one wild type allele. [file 2051-5960-1-18-S3.ppt]

## Slide 1
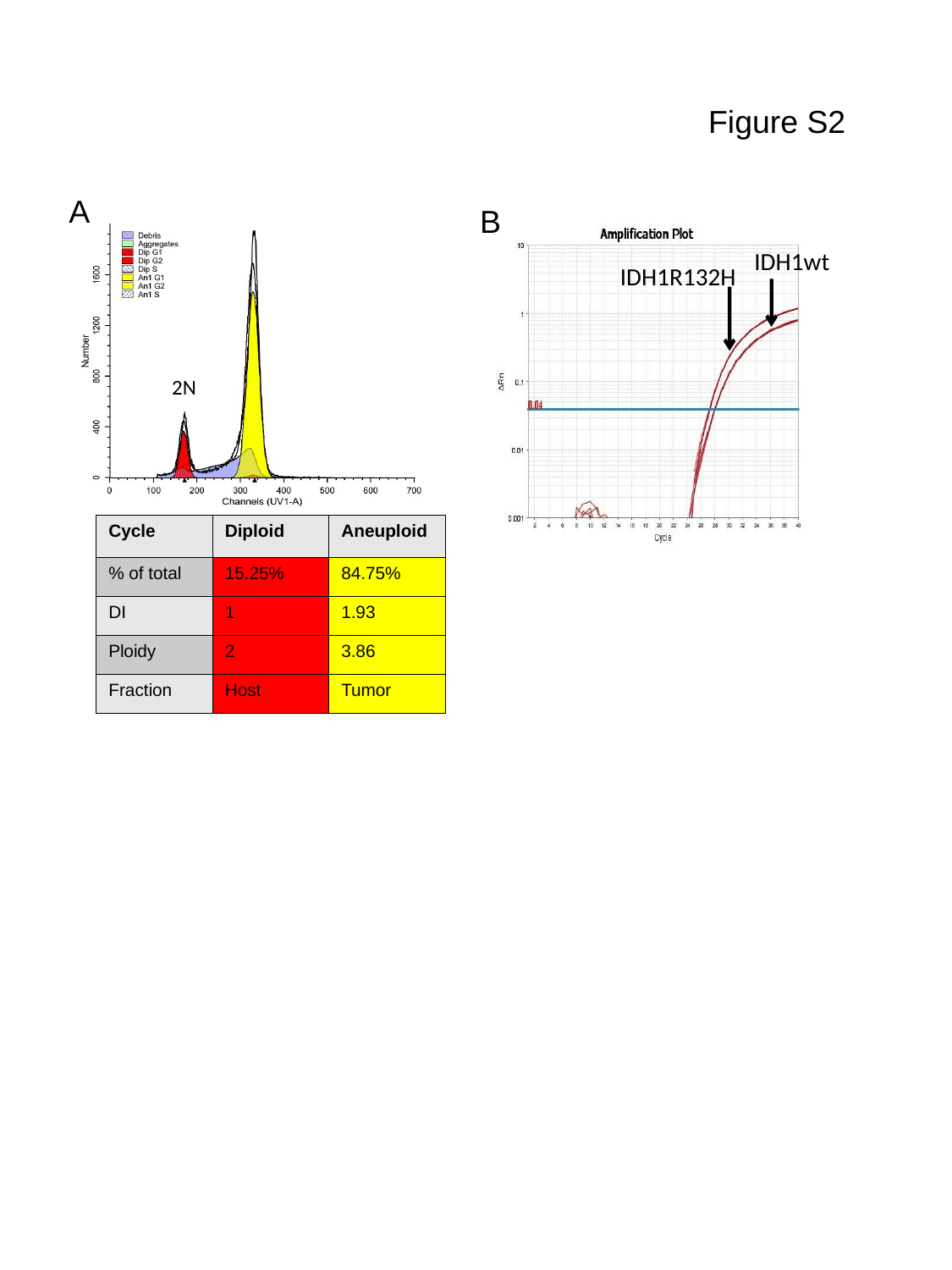

Figure S2
A
B
IDH1wt
IDH1R132H
patient
2N
| Cycle | Diploid | Aneuploid |
| --- | --- | --- |
| % of total | 15.25% | 84.75% |
| DI | 1 | 1.93 |
| Ploidy | 2 | 3.86 |
| Fraction | Host | Tumor |
